# Supplementary material for: Biobased Carbon Dots: From Fish Scales to Photocatalysis
Source: Nanomaterials (Basel). 2021 Feb 18;11(2):524. doi: 10.3390/nano11020524 (PMC7922425; doi:10.3390/nano11020524)
Supplement: Supplementary file 1 [file nanomaterials-11-00524-s001.pdf]

nanomaterials

MDPI

SUPPORTING INFORMATION

Article

Biobased Carbon Dots: From Fish Scales to Photocatalysis

Carlotta Campalani [1], Elti Cattaruzza [1], Sandro Zorzi [1], Alberto Vomiero [1,2], Shujie You [2], Lauren Matthews [3], Marie Capron [3,4], Claudia Mondelli [5], Maurizio Selva [1] and Alvise Perosa [1,*]

[1] Department of Molecular Sciences and Nanosystems, Università Ca' Foscari di Venezia, Via Torino 155, 30172 Venezia Mestre, Italy; carlotta.campalani@unive.it (C.C.); cattaruz@unive.it (E.C.); sandro.zorzi@unive.it (S.Z.); alberto.vomiero@unive.it (A.V.); selva@unive.it (M.S.)
[2] Division of Material Science, Department of Engineering Sciences and Mathematichs, Luleå University of Technology, 971 87 Luleå, Sweden; shujie.you@ltu.se
[3] The European Synchrotron Radiation Facility, 38043 Grenoble Cedex 9, France; lauren.matthews@esrf.fr (L.M.); marie.capron@esrf.fr (M.C.)
[4] Partnership for Soft Condensed Matter PSCM, ESRF The European Synchrotron Radiation Facility, 71 Avenue des Martyrs 38043 Grenoble Cedex 9, France
[5] CNR-IOM, Institut Laue Langevin, 71, Avenue des Martyrs, 38042 Grenoble Cedex 9, France; mondelli@ill.fr
* Correspondence: alvise@unive.it

Table of content

1. ^{1}H, ^{13}C {^{1}H} and two-dimensional (2D) DOSY NMR spectra;
2. FT-IR Spectrum
3. XPS analysis
4. UV-Vis Spectrum
5. PL-PLE Spectrum
6. TIME-RESOLVED PHOTOLUMINESCENCE (PL) measurements
7. SAXS measurements
8. SLS measurements
9. PHOTOCATALITIC EXPERIMENTS - UV-Vis spectrum

1. H, ^{13}C {^{1}H} and two-dimensional (2D) DOSY NMR spectra

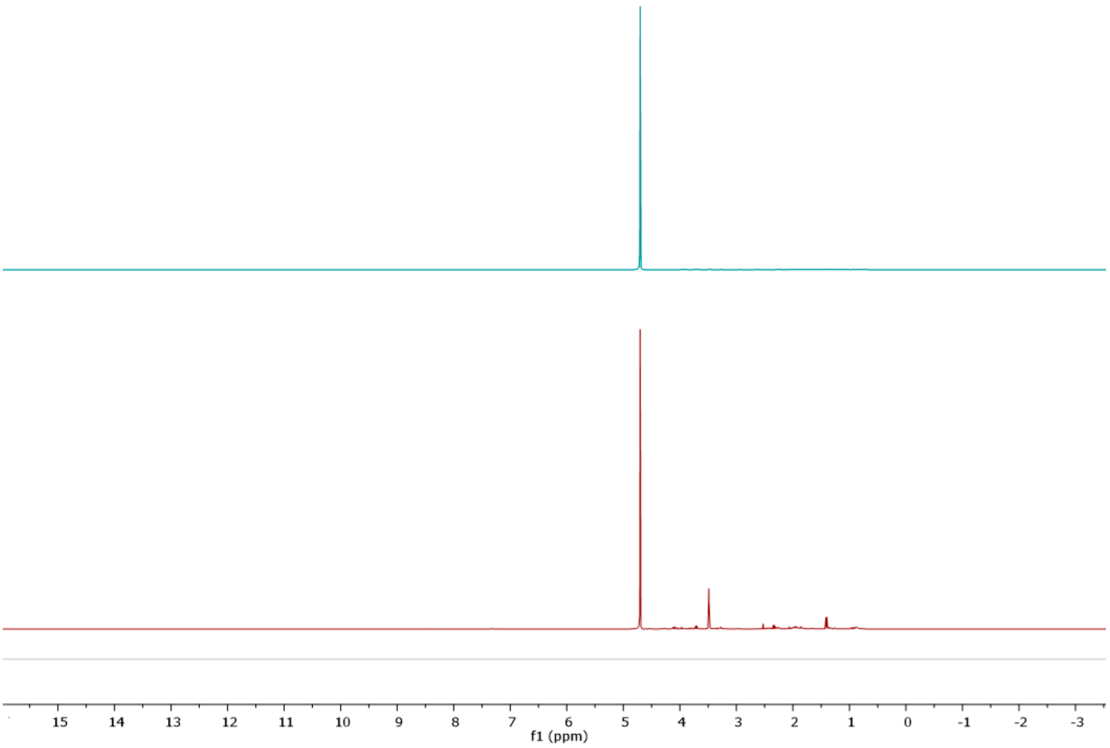

Figure S1. ^{1}H NMR of crude (red line) and purified (blue line) bass-CDs in D_2O.

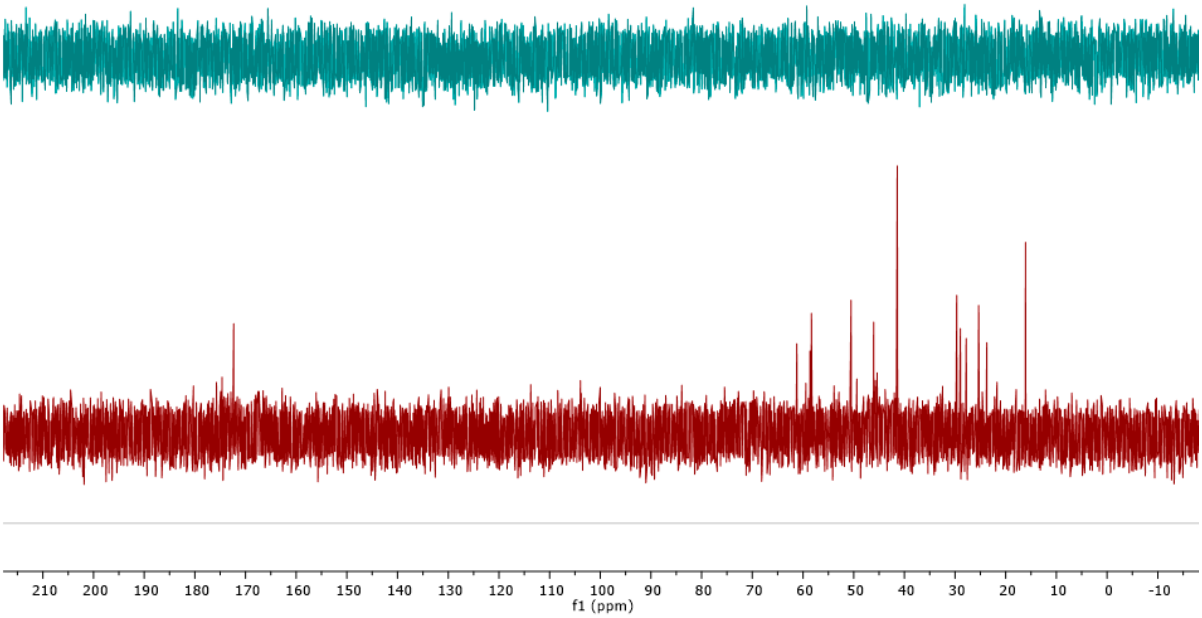

Figure S2. ^{13}C {^{1}H} of crude (red line) and purified (blue line) bass-CDs in D_2O.

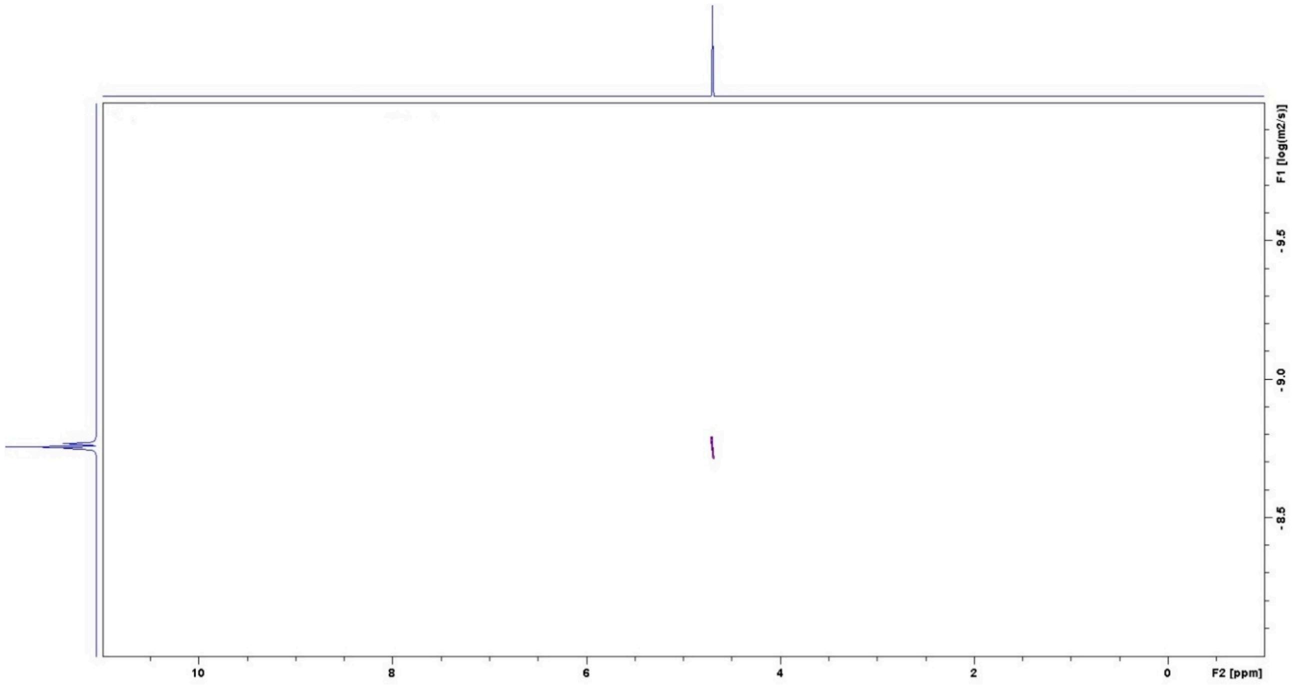

Figure S3. 2D DOSY of purified bass-CDs in D_2O.

2. FT-IR SPECTRUM

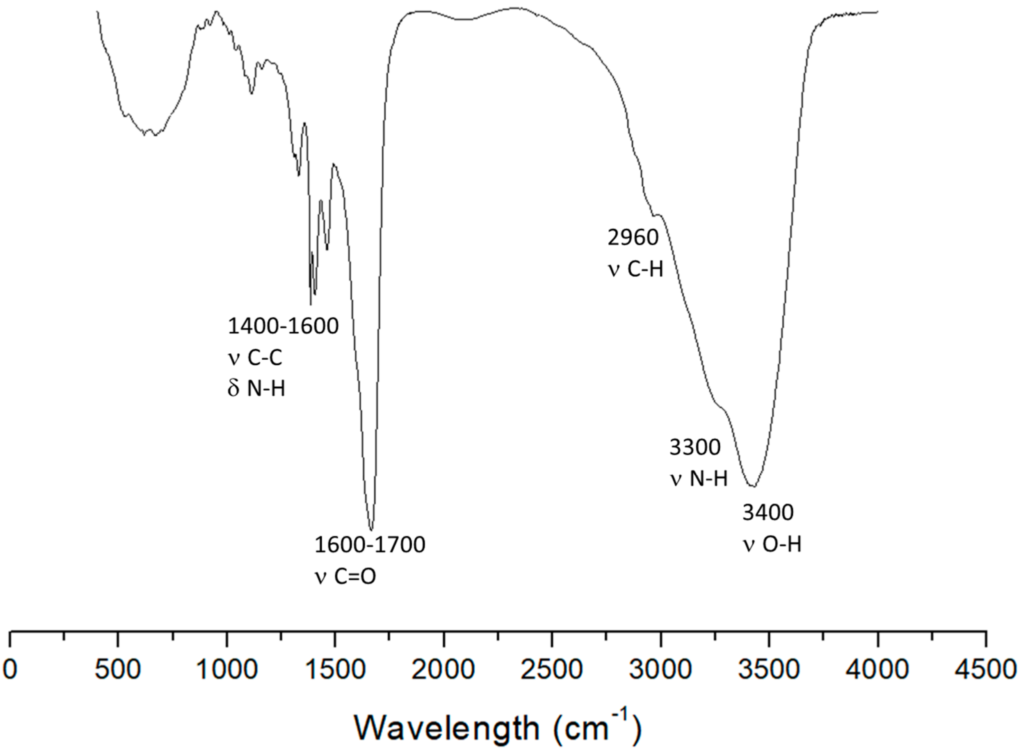

Figure S4. FT-IR spectrum of bass-CDs in KBr.

3. XPS ANALYSIS

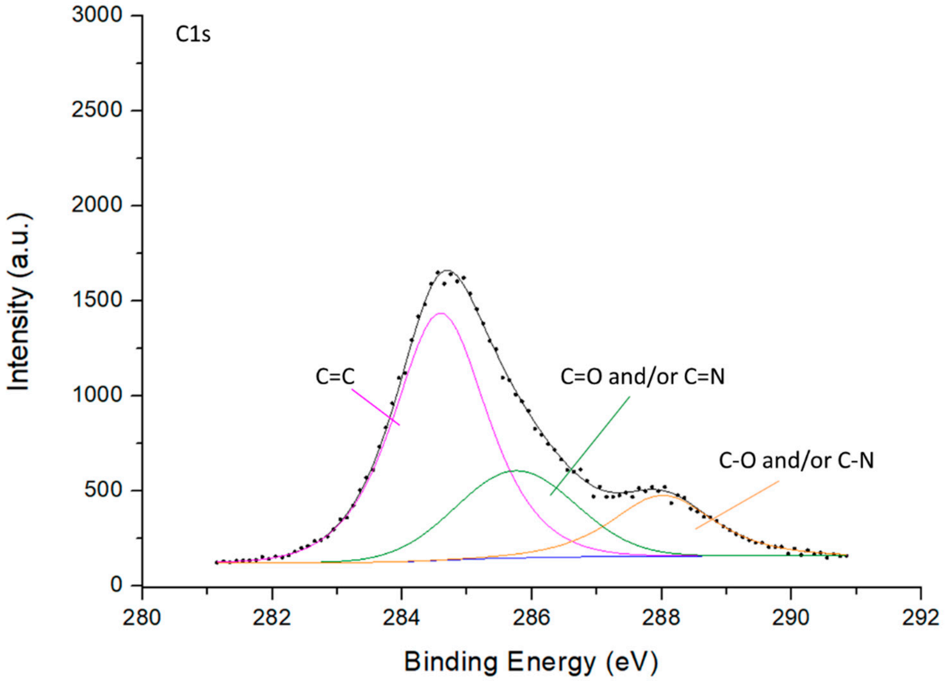

Figure S5. C1s XPS spectrum of bass-CDs.

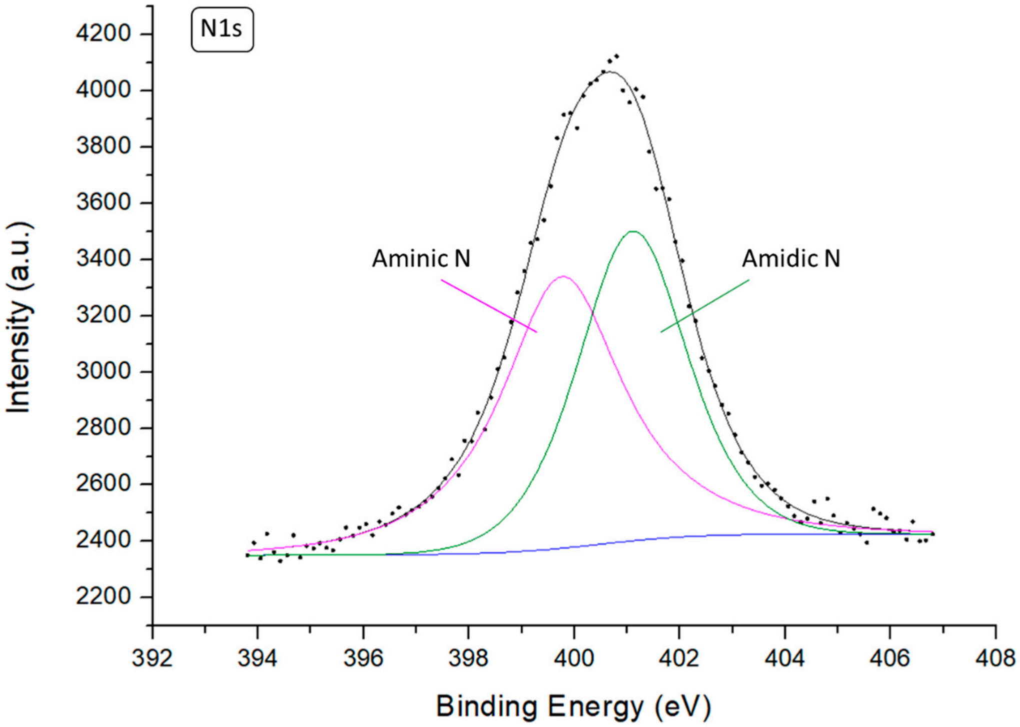

Figure S6. N1s XPS spectrum of bass-CDs.

4. UV-Vis SPECTRUM

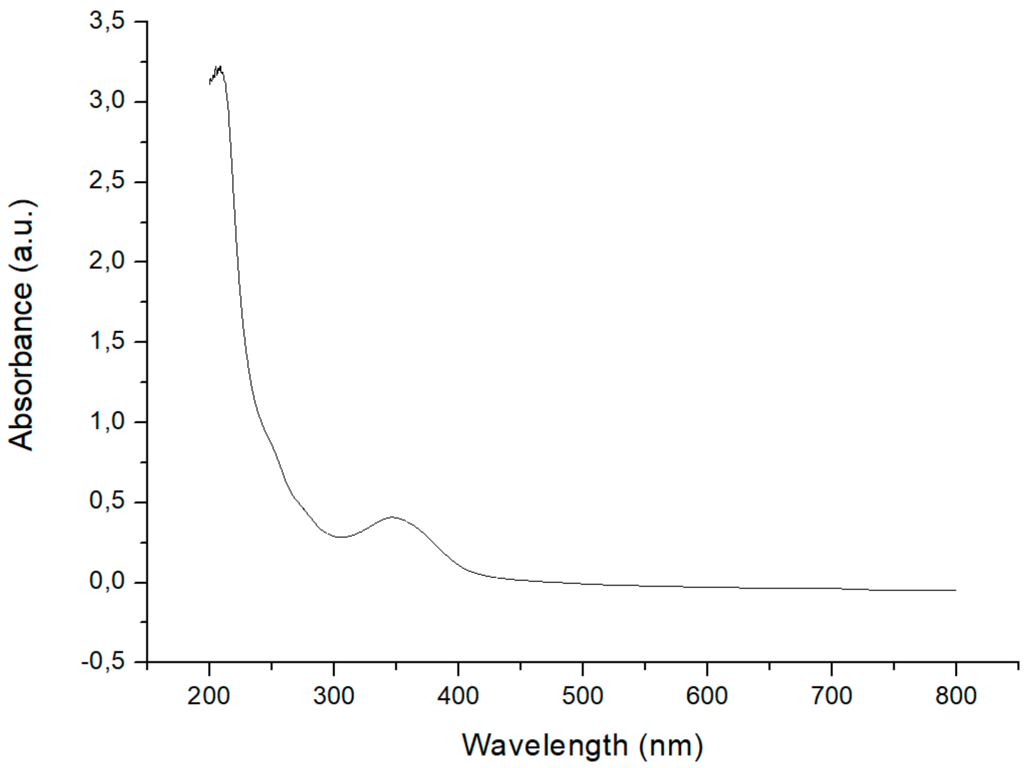

Figure S7. UV-Vis spectrum of bass-CDs.

5. PL-PLE SPECTRUM

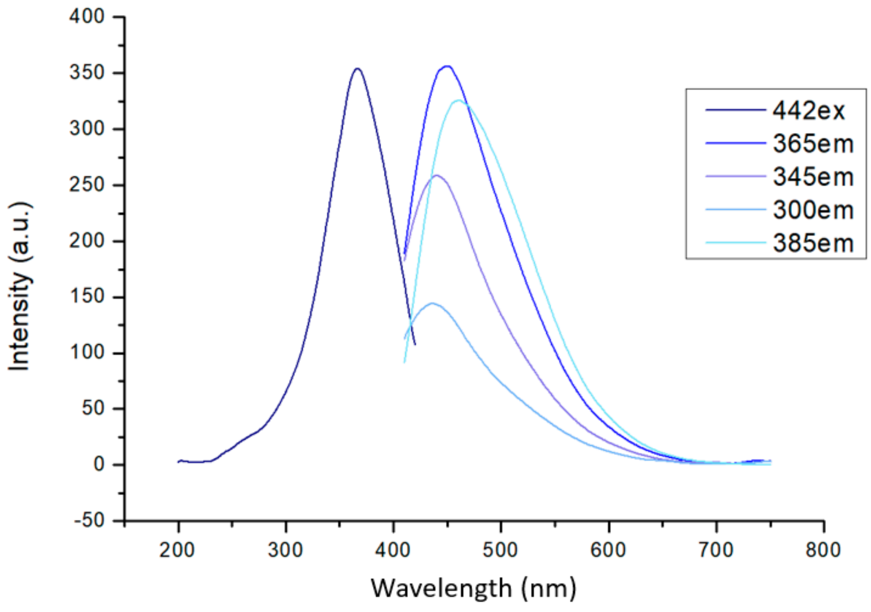

Figure S8. PL-PLE spectrum of bass-CDs.

6. TIME-RESOLVED PHOTOLUMINESCENCE (PL) MEASUREMENTS

The time-resolved PL spectra (Figure 2) were analysed with exponential fitting. Three time-constants were used to fit the emission of synthesized CDs and two time-constants were employed for a-NCDs emission. Bass-CDs has much shorter decay compare to the a-N-CDs sample.

Table S1. Biexponential fitting of lifetime data, excitation = 372 nm. Fitting parameters of photoluminescence decays displayed un figure 2. The average lifetime has been calculated as follows: $\tau_{av} = \frac{B_1\tau_1^2 + +B_2\tau_2^2 + B_3\tau_3^2}{B_1\tau_1 + B_2\tau_2 + B_3\tau_3}$.

	τ_1(ns)	τ_2(ns)	τ_3(ns)	B_1	B_2	B_3	τ_{av}(ns)
bass-CDs	1.151	4.343	12.07	1932	2257	633	6.98
dialyzed bass-CDs	0.873	3.572	11.87	2357	2123	542	6.55
a-N-CDs	6.654	13.89		541	4405		13.5

7. SAXS DATA ANALYSIS

The fitting procedures used in this paper to obtain information regarding the surface roughness of the clusters, have been performed using the SasView software *[ref: 36 principal paper]*.

For fractal structures, the scattering intensity I(Q) express a typical power law behaviour, which in the case of surface fractal structures can be expressed as

$$I(\boldsymbol{Q}) \propto \boldsymbol{Q}^{-(6-D_s)}, \quad with \quad 2 < D_s < 3$$

where $\boldsymbol{Q}$ is the scattering vector and D_s is the surface fractal dimension. For a smooth surface the $D_s = 2$ and the exponent of the power law is equal to 4, while in the case of a rough surface the $D_s = 3$ and the exponent is equal to 3 [ref: https://iopscience.iop.org/article/10.1088/0022-3727/19/8/021/meta]. In the case of mass fractal structures the scattering intensity can be written as

$$I(\boldsymbol{Q}) \propto \boldsymbol{Q}^{-D_m}, \quad with \quad 1 < D_m < 3$$

where D_m is the mass fractal dimension.

In the present work, the surface fractal dimension of the large clusters has been obtained by fitting the low $\boldsymbol{Q}$ region of the SAXS curves with a power law function, contained in the SasView model's library. The signal ascribed to the presence of carbon dot aggregates in the Bass-CDs sample, has been described using a power law model, which has highlighted its mass fractal behaviour and was promptly described with a mass fractal model. In this model the $\mathrm{I}(\boldsymbol{Q})$ is mostly described by a spherical form factor $\mathrm{P}(\boldsymbol{Q})$, that takes into account the scattering of the primary particles with radius R composing the clusters. The structure factor, $\mathrm{S}(\boldsymbol{Q})$, is well described elsewhere [ref same as before: https://iopscience.iop.org/article/10.1088/0022-3727/19/8/021/meta], taking into account the mass fractal behaviour of the clusters, characterized by the cluster size ξ, and the mass fractal dimension D_m. For the fitting procedure, we used the cluster size obtained from SLS measurements (120 ± 30) nm. The scattered intensity $\mathrm{I}(\boldsymbol{Q})$ for the a-N-CDs has been described by a power law function, taking into account the scattering arising from the large aggregates. The precise cluster size cannot be obtained due to the fact that only the tail of their scattering contribution in the low Q region is seen in the SAXS curves. For the Bass-CDs sample, the $\mathrm{I}(\boldsymbol{Q})$ has been described by a sum of functions: the mass fractal model and the power law function, in order to compute the intensity originating from the smaller aggregates. In fig. S9 the best fits for the a-N-CDs and Bass-CDs are reported. There are some visible fluctuations in the curve between ~ 0.002 – 0.005 Å^{-1}, it is likely that, rather than real oscillations, this is a result of an uncertainty in the background subtraction. Where the data is similar in intensity to the water background and the oscillations are a result of noise in the sample scattering. The background in the high $\boldsymbol{Q}$ region of the scattering profile is not flat as expected, this is likely a result of the water background subtraction, which does not fully compensate the background of the sample. Despite this, the low signal in the high $\boldsymbol{Q}$ region indicates that no scattering other than the background was observed. The parameters of the best fit are reported in tab. S2.

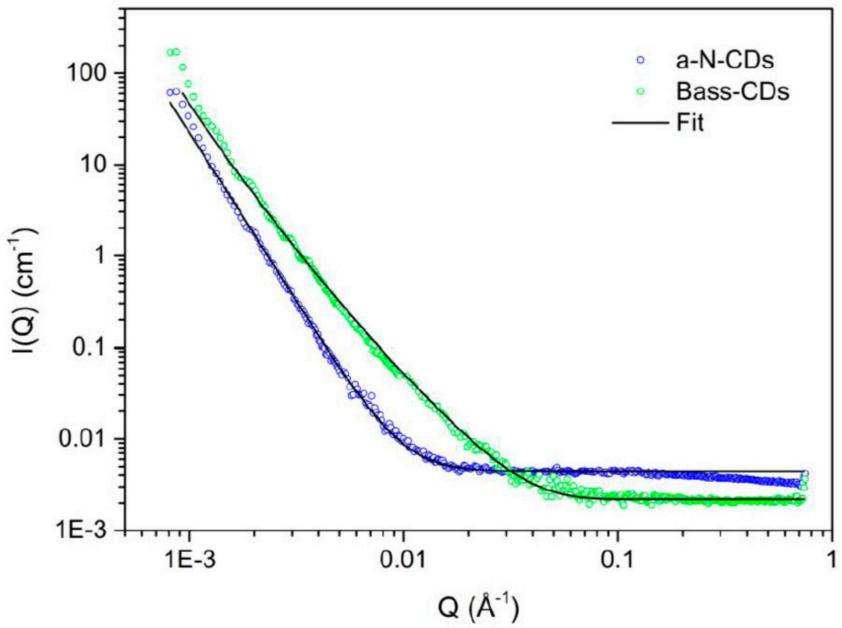

Figure S9. Scattering intensity *I(Q)* vs *Q* for both CDs samples, together with the best curve fit obtained by using the model described in the text. Blue dots are related to the SAXS data of the a-N-CDs sample, green dots for the Bass-CDs and the solid black line is for the fit curves.

Table S2. List of the parameters of the fit curves reported in Figure **S9**. * The values between brackets are the surface fractal dimensions D_S.

sample	Power Law model	Mass Fractal model		
	Exponent*	Mass fract. dim D_m	Radius R (nm)	Cluster size ξ (nm)
Bass-CDs	3.86 ± 0.05 (2.14 ± 0.05)	2.3 ± 0.1	4 ± 1	127 ± 8
a-N-CDs	3.71 ± 0.05 (2.29 ± 0.05)			

8. SLS MEASUREMENT

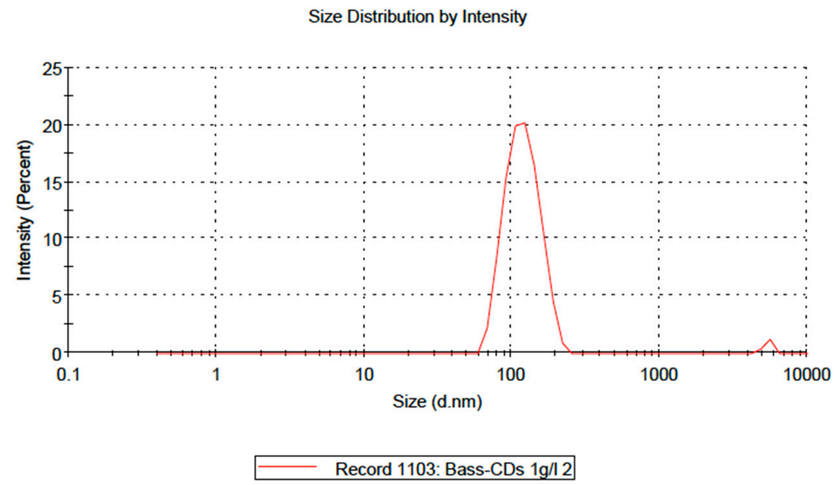

Figure S10. SLS size distribution vs intensity in percent. The average size of the clusters is (120 ± 30) nm.

9. PHOTOCATALYTIC EXPERIMENT – UV-Vis SPECTRUM

A solution composed by EDTA, MV and CDs (concentrations and conditions reported in the main text) were placed under inert atmosphere in a quartz cuvette. The solutions were then irradiated at 365 nm fixed wavelength emission (Hangar s.r.l.; ATON LED-UV 365; 80 W/m2 of irradiance in the UVA spectral range 315-400 cm^{-1}). The progress of the reactions was monitored using an UV spectrophotometer following the formation of the typical absorption band of the reduced $MV^{+\cdot}$ radical cation form centred at 605 nm and its concentration was estimated using ε = 13700 M^{-1} cm^{-1} (Figure S11).

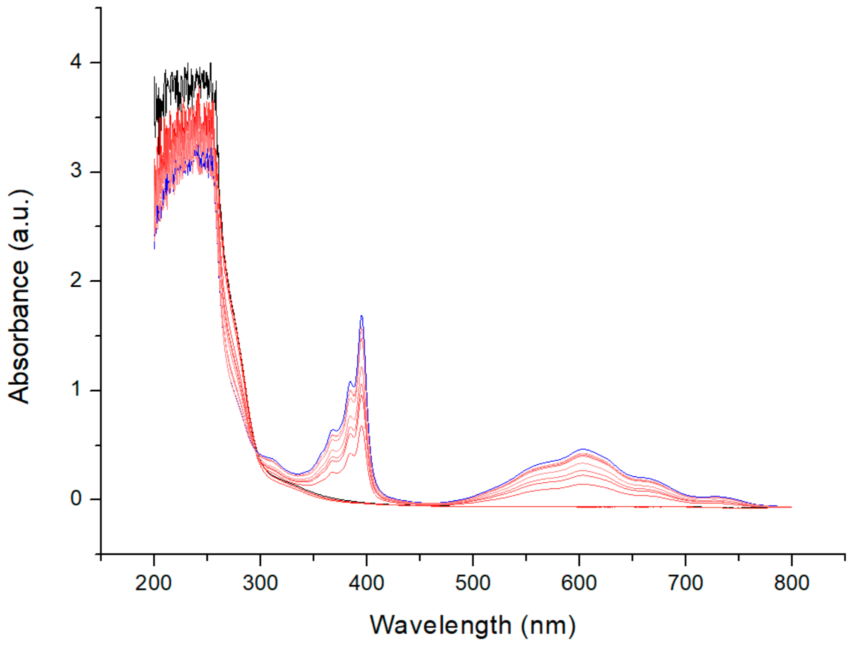

Figure S11. Time resolved UV-Vis spectra of bass-CDs (0.2 mg/mL) with methyl viologen (MV^{2+}, 60μM) in aqueous EDTA (0.1 M) under inert atmosphere. (blue line after 30 minutes of reaction).
